# Alcoholic vs non-alcoholic fatty liver in rats: distinct differences in endocytosis and vesicle trafficking despite similar pathology

**DOI:** 10.1186/s12876-016-0433-4

**Published:** 2016-02-29

**Authors:** Karuna Rasineni, Daniel D. Penrice, Sathish Kumar Natarajan, Mark A. McNiven, Benita L. McVicker, Kusum K. Kharbanda, Carol A. Casey, Edward N. Harris

**Affiliations:** Department of Internal Medicine, University of Nebraska Medical Center, Omaha, NE USA; The Liver Study Unit, VA Nebraska-Western Iowa Health Care System, Omaha, NE USA; Department of Biochemistry and Molecular Biology, University of Nebraska Medical Center, Omaha, NE USA; Department of Biochemistry and Molecular Biology and the Center for Digestive Diseases, Mayo Clinic, Rochester, MN USA; Department of Biochemistry, University of Nebraska-Lincoln, Lincoln, NE USA; Dept. of Nutrition and Health Sciences, University of Nebraska-Lincoln, Lincoln, NE USA

**Keywords:** Alcoholic fatty liver disease (AFLD), RabGTPase proteins, Asialoglycoprotein receptor (ASGPR), Receptor-mediated endocytosis, Non-alcohol fatty liver disease (NAFLD)

## Abstract

**Background:**

Non-alcoholic and alcoholic fatty liver disease (NAFLD and AFLD, respectively) are major health problems, as patients with either condition can progress to hepatitis, fibrosis, and cirrhosis. Although histologically similar, key differences likely exist in these two models. For example, altered content of several vesicle trafficking proteins have been identified in AFLD, but their content in NAFLD is unknown. In this study, we compared select parameters in NAFLD and AFLD in a rat model.

**Methods:**

We fed either Lieber- DeCarli liquid control or alcohol-containing (35 % as calories) diet (AFLD model) or lean or high-fat (12 or 60 % derived from fat, respectively) pellets (NAFLD model) for 8–10 weeks, n = 8 in each model. Serum, hepatocytes and liver tissue were analyzed. Liver injury markers were measured in serum, triglyceride content and endocytosis (binding and internalization of ^125^I- asialoorosomucoid) was measured in isolated hepatocytes, and content of selected trafficking proteins (Rab3D, Rab7 and Rab18) were determined in whole liver tissue.

**Results:**

Although liver injury markers and triglyceride content were similar in both models, binding and internalization of ^125^I- asialoorosomucoid was significantly impaired in the hepatocytes from AFLD, but not NAFLD, animals. In addition, protein content of the asialoglycoprotein receptor (ASGPR) and three trafficking proteins, Rab3D, Rab7and Rab18, were significantly decreased after alcohol, but not high-fat feeding. Levels of protein carbonylation, amount of glutathione stores, and lipid peroxidation were similar irrespective of the insult to the livers that resulted in fatty liver.

**Conclusion:**

Impairments in protein trafficking in AFLD are likely a direct result of alcohol administration, and not a function of fatty liver.

## Background

Fatty liver disease is a prevalent health risk in modern society, can arise from a variety of etiologies, and can progress to hepatitis, fibrosis, and cirrhosis. A triglyceride content of greater than 5 % in the liver is defined as a fatty liver [1], and there are two primary types: alcoholic fatty liver disease (AFLD) and non-alcoholic fatty liver disease (NAFLD). The first is caused by the consumption of alcohol (ethanol is oxidized by alcohol dehydrogenase to form acetaldehyde which feeds into the acetyl-CoA pathway and, when in excess, undergoes synthesis of long chain fatty acids) to form triglycerides in hepatocytes [2]. The second is the over-consumption of a high fat, high sugar diet in which excess fat is stored in the liver [3]. NAFLD as a result of a high fat/high sugar diet, also called a Western diet, is very prevalent (20–30 % in US population) and it is projected to be the #1 causation for liver transplantation in less than 7 years [4]. Indeed, NAFLD afflicts 85–90 % of overweight/obese individuals of the US [5].

Fatty liver arising from either alcohol consumption or the over-consumption of a high fat/high sugar diet has a similar phenotype in that the hepatocytes accumulate triglycerides. Additionally, both are known to result in increased injury markers, such as increased serum alanine aminotransferase (ALT), aspartate aminotransferase (AST) and alkaline phosphatase (ALP). Of interest to our laboratories is that while there is extensive data from a variety of laboratories on the biochemical alterations present in the liver during AFL, little data is present on any of these parameters in NAFLD models. Previous studies have focused on the effects of ethanol exposure which inhibits clathrin-mediated endocytosis by inhibition of plasma membrane fusion [6] and more specifically on the asialoglycoprotein receptor (ASGPR) trafficking and ligand interactions in our lab [7, 8]. Additionally, we have studied several small Rab-GTPases known to be involved in vesicle trafficking and show decreased content in livers of AFLD animals [9]. In the present study we asked whether these impairments in ASGPR function and Rab-GTPase content in AFLD would also occur in the setting of NAFLD.

## Methods

Ethanol was purchased from Pharmaco-AAPER (Brookfield, CT). IRDye infrared secondary antibodies (Abs) and blocking buffer were from Li-COR Biosciences (Lincoln, NE). Protease inhibitor cocktail (P2714-1BTL), phosphatase inhibitor (p0044), collagenase (type IV), human orosomucoid (α1-acid glycoprotein), and mouse anti-Rab7 antibody were obtained from Sigma (St. Louis, MO). The rabbit polyclonal anti-ASGPR antibody was produced in Dr. Casey’s laboratory and has been described previously [10]. Goat anti-Rab 18 antibody was from Santa Cruz Biotechnology (Santa Cruz, CA). Mouse anti-perilipin 2 (anti-PLIN2) antibody was from Fitzgerald (Acton, MA). Mouse anti-actin antibody was obtained from Millipore (Billerica, MA). Rab 3D polyclonal antibody was kindly provided by Mark A. McNiven (Mayo Clinic, Rochester, MN). PureLink RNA Mini Kit and TaqMan Gene Expression Master Mix were purchased from Life Technologies (Grand Island, NY). All other chemicals were obtained from Sigma Chemical Co. unless stated otherwise.

### Animals, diet administration, hepatocyte isolation and tissue collection

All animals received humane care in accordance with the guidelines established by the American Association for the Accreditation of Laboratory Animal Care (AAALAC) and Animal Research: Reporting of In Vivo Experiments (ARRIVE) [11] . All experimental procedures and ethical standards involving animals were reviewed and approved by the Institutional Animal Care and Use Committees at the Veteran’s Administration Nebraska-Western Iowa Health Care System Research Service (IACUC #11-067-07, approved on Aug. 24, 2014), site of the AFLD studies and University of Nebraska Life Sciences Annex (IACUC #956, approved on Oct. 23, 2013), site of the NAFLD studies. Male Wistar rats weighing 175 to 200 g were divided into two groups (AFLD and NAFLD) with 8 pairs in each group to get statistically relevant data (Student’s paired *t* Test). As described previously [12] for the AFLD model, rats were pair-fed with control or EtOH-containing Lieber–DeCarli diets [13] contained 18 % of total calories from protein, 35 % from fat, 11 % from carbohydrate, and 36 % from ethanol. In the control diet, ethanol was replaced isocalorically with maltodextrin. For the NAFLD model, rats were allowed *ad libitum* access to pellet diet and water. Rats in the HFD group (ResearchDiets #D08060104) were fed a diet with a caloric formulation of 60 % calories derived from fat (lard; a mixture of mono-, poly- and unsaturated fatty acids), 20 % from carbohydrates (corn starch, maltodextrin), and 20 % from protein (Casein) and rats fed a lean diet (Research Diets #D12450K with a caloric (kcal) composition of 10.0 % derived from fat (lard), 70 % from carbohydrates (corn starch, maltodextrin) and 20 % from protein (Casein). Both NAFLD and AFLD diets were similar for protein and carbohydrate content with a special care of avoiding sucrose and fructose. These diets have been proven to induce AFLD and NAFLD symptoms and disease that model human alcohol induced and non-alcohol induced fatty liver, respectively. Rats in both alcohol and high fat-diet groups were housed at AAALAC certified institutions (Omaha VAMC and UNL) in approved housing facilities and transported to the laboratories for terminal surgical procedures. Rats in the NAFLD groups were fed *ad libitum* throughout the study; rats in the AFLD group were fed the ethanol diet *ad libitum*, and the control rat received an equivalent amount of diet that its pair-fed ethanol consumed. On the day of sacrifice, control animals were meal-fed, to minimize variations in feeding patterns [15]. Rats were sacrificed in the morning hours eight weeks after the initiation of the diet regimen after being anaesthetized with 4 % isoflurane gas mixed with oxygen in a 0.9 m^3^ chamber. Blood samples were collected via the axillary artery, and serum used for analysis. A piece of whole liver was obtained for histological observation (after knotting a small portion of a small liver lobe with surgical polyester thread); the remaining liver was perfused with isotonic buffers containing collagenase (Type 1 V, Sigma #C5138) as described previously [14, 15].

### Clinical chemistry

The serum profile was measured by a Vetscan chemistry analyzer (Abaxis, Union City, CA). Serum samples were loaded on the Mammalian Liver Profile reagent rotor and read with VetScan VS2 Chemistry Analyzer. The Mammalian Liver Profile reagent rotor provided the quantitative measurements of alanine aminotransferase (ALT), alkaline phosphatase (ALP), albumin (ALB), bile acids, and total cholesterol.

### Liver histology

Paraffin-embedded liver tissue sections were processed for hematoxylin/eosin staining and evaluated for steatosis and inflammation.

### Immunohistochemistry

Immunohistochemical staining for lipid droplet protein (PLIN2) was performed as described previously [9]. Briefly, paraffin-embedded liver sections were deparaffinized in xylene and rehydrated in ethanol. Following deparaffinization, slides were subjected to antigen retrieval process with 10 mM sodium citrate buffer (pH 6) for 20 min. Sections were incubated overnight with PLIN2 antibody and followed by staining with appropriate Alexa Fluor secondary antibody. Sections were mounted with vectashield mounting medium containing DAPI. Confocal images were acquired using a Zeiss 510META laser scanning confocal microscope.

### Triglycerides

The extraction of lipids from hepatocytes was carried out according to the procedure of Folch and colleagues [16]. Aliquots of lipid extract were saponified to quantify the triglycerides (TGs) using the TG diagnostic kit (Thermo dimethyl adipimidate (DMA) kit; Thermo Electron Clinical Chemistry, Louisville, CO).

### Western blot analysis

Liver homogenate (20 %) was prepared in 60 % sucrose in TE buffer (10 mM Tris–HCL, 1 mM EDTA, pH 7.4) containing a protease and phosphatase inhibitor cocktail (Sigma, St. Louis, MO). Liver post-nuclear supernatant (PNS) fractions were obtained by centrifugation (1000 × g) of the homogenate for 10 min. Liver PNS samples were separated by 12 % SDS-PAGE, blotted on nitrocellulose and proteins were detected with appropriate primary antibodies and then immunoreactive proteins were visualized and quantified using the Odyssey Infrared Imager and associated software.

### Preparation and labeling of asialoorosomucoid (ASOR)

Human orosomucoid (Sigma, St. Louis, MO) was desialylated by the neuraminidase procedure as described by Oka and Weigel [17]. ^125^I-ASOR was prepared by the procedure described previously [15]. Briefly, 125 μg of ASOR in 200 μl of PBS was reacted with 38 μg 1,3,4,6-tetrachloro-3α,6α-diphenyl-glycoluril dried oxidizing reagent coated on the bottom of a glass tube and 0.3 mCi Na^125^I at room temperature for 15 min. The labeled ASOR was then separated from unincorporated Na^125^I with a PD-10 column and quantified by the Bradford assay.

### Measurements of ASOR binding and internalization

Assessment of internalization of ASOR was determined by a modification of our previous studies [15]. Briefly, hepatocytes isolated from AFLD and NAFLD animals were plated in triplicate on collagen coated 24-well plates at a concentration of 10^5^ cells/well and incubated at 37 °C with 1.0 μg/mL ^125^I-ASOR (25nM) for various periods of time (0, 30, 60, 90 & 120 min). Receptor specificity was measured in replicate wells containing excess unlabeled ASOR to assess background levels. After each incubation period, cells were washed with phosphate-buffered saline and the amount of radioactive ligand in cell lysates was determined and normalized to total cell lysate protein.

### mRNA analysis /Analysis of gene expression

For quantification of mRNA, RNA was isolated from liver pieces using a PureLink RNA Mini Kit (Invitrogen, Carlsbad, CA) and was reverse transcribed from 1 μg of total RNA using oligo-dT primers and the Transkriptor kit (Roche Applied Science). To determine gene expression levels, real-time PCR reactions were performed using rat-specific primers from the TaqMan Gene Expression Assay System (Rab3D; catalog # rn00756153; Rab 7, catalog # rn00592246; Rab18, catalog # rn01526466; ASGPR, catalog # rn00560750) and samples were analyzed in the 7500 Real Time PCR System (Applied Biosystems, Carlsbad, CA). The ∆∆Ct method was used to determine the fold change using actin for normalization.

### Serum hormone quantification

Metabolic hormones; leptin, amylin and insulin were measured using the Multiplex MAP Magnetic Bead-based immunoassay kits (Millipore Corp. Billerica, MA). The assay was conducted according to the manufacturer’s instructions using handheld magnetic separator block for 96-well flat bottom plates (Millipore, Millipore Corp) and analyzed using the Luminex 200 system (Luminex Corp., Austin, TX). All samples were run in duplicate and standards supplied by the manufacturer were run on each plate. Mean fluorescence intensity was analyzed using the BioPlex manager software version 5.0 (Bio-Rad, Hercules, CA) and compared to a standard curve to calculate the concentrations. Values below the range of the standard curve were set to the lower limit of detection.

### Selected oxidative stress-related parameters in liver tissue

Pro-oxidant formation was assessed by measuring protein carbonyl content as described [18] using 2, 4-dinitrophenylhydrazine and calculated using an extinction coefficient of 22 mM^−1^ cm^−1^. In addition, the extent of lipid peroxidation was assessed by quantitating thiobarbituric acid reactive substances (TBARS) following the procedure of Uchiyama & Mihara [19] using malondialdehyde (MDA) as a standard. Anti-oxidant defense was quantified by measuring total and oxidized glutathione (GSSG) using the enzymatic method [20].

### Statistical analysis

The results are expressed as mean ± SEM. Comparison between control and their respective experimental animals was analyzed using the Student’s *t*-test. p-values of <0.05 were considered significant.

## Results

### Morphology of liver tissue in alcohol and non-alcohol induced fatty liver

**Fig. 1:**
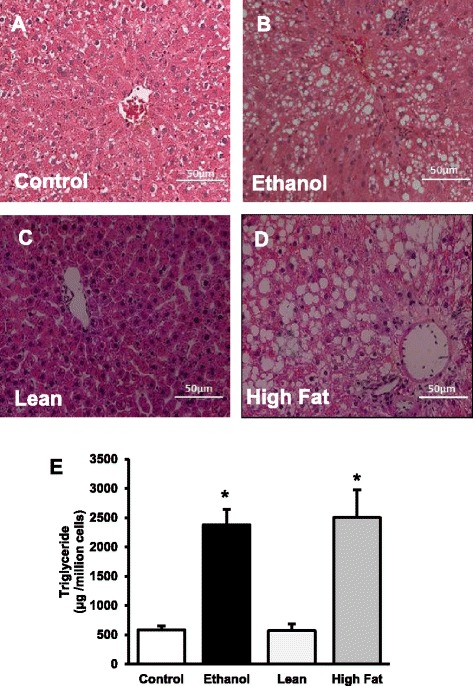
Triglyceride accumulation in the hepatocytes of control and EtOH-fed rats (**a, b**) and of lean and HFD rats (**c, d**). Hematoxylin and Eosin staining was performed on paraffin sections. Images are representative of each pair-fed group of *n* = 8. Magnification, 200×. **e** Quantification of lipid content in isolated hepatocytes. Hepatocyte TGs were extracted with chloroform: methanol (2:1) and amount of glycerol were measured in each sample. TG levels were significantly increased in both AFLD and NAFLD rats when compared to their respective controls. Values are means ± SEM, *n* =8, *p ≤ 0.05

**Fig. 2:**
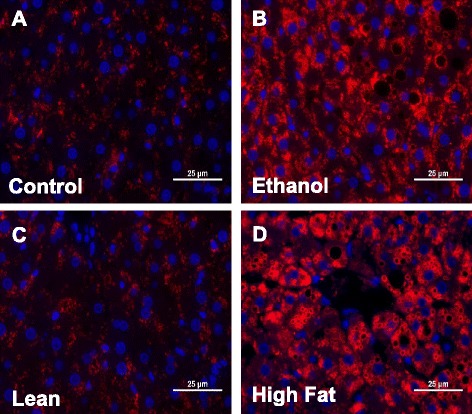
PLIN2 staining for lipid droplets: Liver sections from Control and EtOH-fed (**a**, **b**) and Lean and HFD(**c**, **d**) rats both show increased lipid droplet accumulation (as identified by PLIN2) when compared to their controls. Images are representative of each pair-fed group of *n* = 8. Magnification, 400×

At the outset of these experimental procedures, we confirmed those rats on the Lieber-DeCarli (EtOH) diet and their cohorts on the high fat diet (HFD) had fatty liver by H&E staining of the liver tissue (Fig. 1). The increased fat content was also quantitated by measuring triglyceride content in purified hepatocytes. The amount of triglyceride was similar between the control groups for both AFLD and NAFLD animals, and in the alcohol-fed and high-fat groups, indicating that induction of fatty liver by both diets were equivalent (Fig. 1e). PLIN 2 staining (a lipid droplet marker) also revealed increases in LD accumulation between controls and experimental groups (Fig. 2).

### Comparative serum profiles in NAFLD and AFLD

**Fig. 3:**
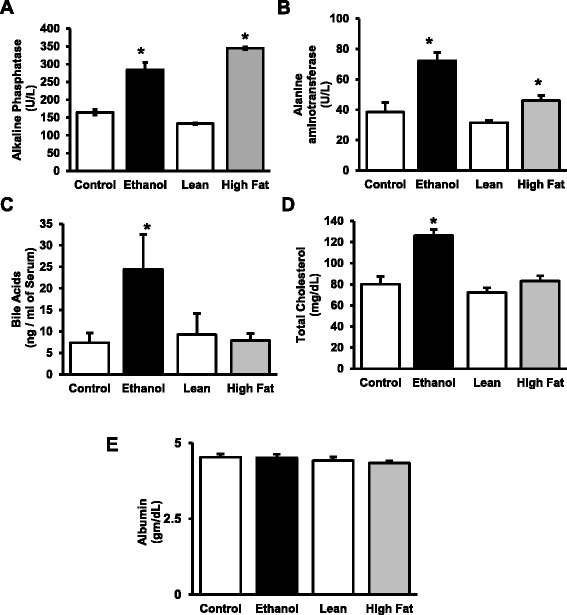
Liver markers in serum of AFLD/NAFLD rats. The VetScan Liver Profile system for analyzing serum revealed specific changes in enzyme and molecular markers as indicated. The EtOH-fed and HFD cohorts were compared against their respective dietary controls. Values are means ± SEM, *n* =8, *p ≤ 0.05

**Fig. 4:**
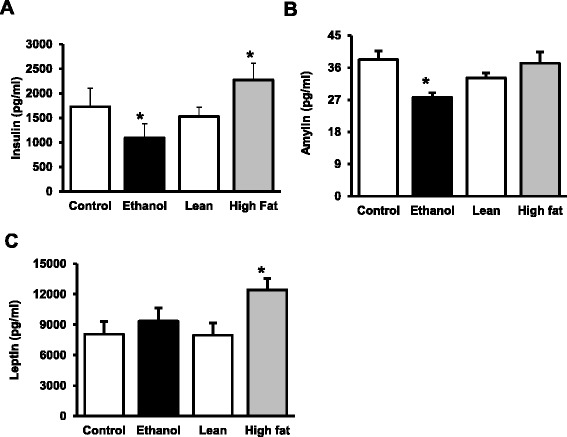
Metabolic markers in serum AFLD/NAFLD rats. Serum from AFLD and NAFLD rats was analyzed by the Milliplex Map Rat Metabolic Bead Panel (Millipore), and compared against their respective dietary controls. Values are means ± SEM, *n* =8, *p ≤ 0.05

Serum from each animal was tested for markers of liver damage using the Liver Profile Rotorary disc (Abaxis). We measured alkaline phosphatase (ALP) is a marker for a number of disorders including blocked bile ducts [21] and some forms of cancer [22], but in our models, it is a sign of cholangiocyte stress and/or damage. ALP levels in the experimental animals (EtOH-fed or HFD) were significantly higher than the matched controls (Fig. 3a). Alanine aminotransferase (ALT) is an indicator of hepatocyte cell death [23] was also increased in both the high-fat groups (Fig. 4b). Bile acids (Fig. 3c) and cholesterol (Fig. 3d) were significantly higher in the EtOH-fed rats, but no significant differences were identified between high-fat and lean controls in NAFLD model. These results suggest that the synthesis of lipids from alcohol metabolic precursors has a greater impact on cholesterol biosynthesis than the absorption of fatty acids and esterification of triglycerides [24, 25]. Levels of albumin, an abundant serum protein produced by hepatocytes, were not affected in either model (Fig. 3e).

We also analyzed pancreatic hormones level in serum using the Rat Metabolic Hormone Panel provided by Milliplex systems. Insulin resistance has been shown to be associated with NAFLD/AFLD [26–28] and, not surprisingly, our NAFLD model demonstrated hyperinsulinemia (Fig. 4a). In contrast, despite similar glucose levels observed in both control and ethanol rats in the fed condition (data not shown); insulin levels in the serum of EtOH-fed rats were lower than the controls. In NAFLD animals, along with increased insulin in the serum (Fig 4a), we also observed increased glucose (data not shown). These values were obtained in fed animals, and further studies on insulin resistance and determination of HOMA-IR will be important to examine in fasted animals. Like insulin, amylin is a peptide hormone produced by pancreatic beta cells and is co-secreted with insulin to decrease gastric emptying and increase satiety. Levels of amylin were unchanged in HF-fed rats suggesting that pancreatic beta cells are normal. In AFLD rats, the levels were significantly decreased in the EtOH-fed rats compared to the dietary control (Fig. 4b). The satiety hormone, leptin, was increased in NAFLD rats and unchanged in AFLD rats (Fig. 4c). These results may be reflective of the administration of the diet which is *ad libitum* for NAFLD and calorie restricted for the AFLD diet or the effects of ingested alcohol on pancreatic function.

### Receptor-mediated endocytosis in AFLD versus NAFLD

**Fig. 5:**
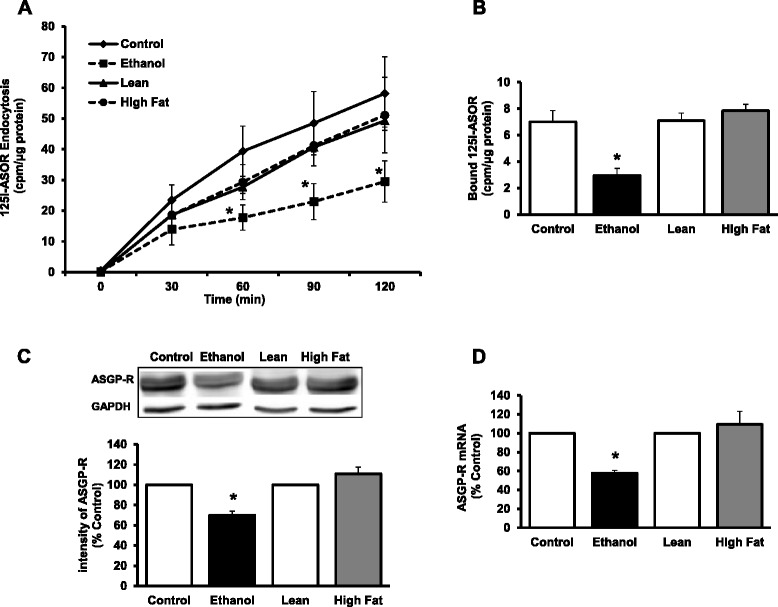
ASGPR mediated endocytosis in fatty liver. **a** Endocytosis of ASOR. Hepatocytes were allowed to internalize ^125^I-ASOR at 37 °C and cell samples were collected at the indicated time points, washed, and radioactivity and protein content determined. Results were calculated by the means ± SEM, *n* = 4. Values compared against their respective dietary controls. p* < 0.05. **b** Binding of ^125^1-ASOR to hepatocytes. Hepatocytes were incubated at 0 °C for 60 min with 1 μg/mL ^125^1-asialo-orosomucoid; specific binding was determined in the presence of 100 fold XS of cold ASOR. Data are expressed as CPM/μg protein for the various groups and are means ± SEM, *n* =8; *p ≤ 0.05. **c** Quantification of ASGP-R protein in liver. Liver lysates were subjected to WB analysis and quantitative data obtained after normalization with GAPDH. **d** The mRNA levels of ASGP-R were measured by qPCR analysis and data were presented after normalization with β-actin. Values represent the fold change compared to respective controls

Next, we focused on hepatocyte endocytosis in the isolated primary cells from the two models. Our previous work has shown that alcohol impairs multiple aspects of the process of receptor-mediated endocytosis (RME), using the ASGPR as a model (8–11, 15). In the present studies, we used a radiolabeled ligand (^125^1-Acid glycoprotein or orosomucoid (ASOR)), we found that ASGPR internalization rates with cargo in AFLD are 50 % of the control (Fig. 5a), in contrast to NAFLD model in which endocytosis rates are unchanged between control and HF-fed rats. Follow-up binding studies at 4 °C, indicate that the level of surface receptors is lower in AFLD hepatocytes (Fig. 5b), but not in NAFLD hepatocytes. ASGPR protein and mRNA levels in the AFLD rats were decreased by about 30 and 45 %, respectively. In NAFLD rats, ASGPR levels were slightly increased as compared with the control rats, but these differences were not statistically distinguishable (Fig. 5c & d).

### Vesicle protein trafficking defects in AFLD versus NAFLD

**Fig. 6:**
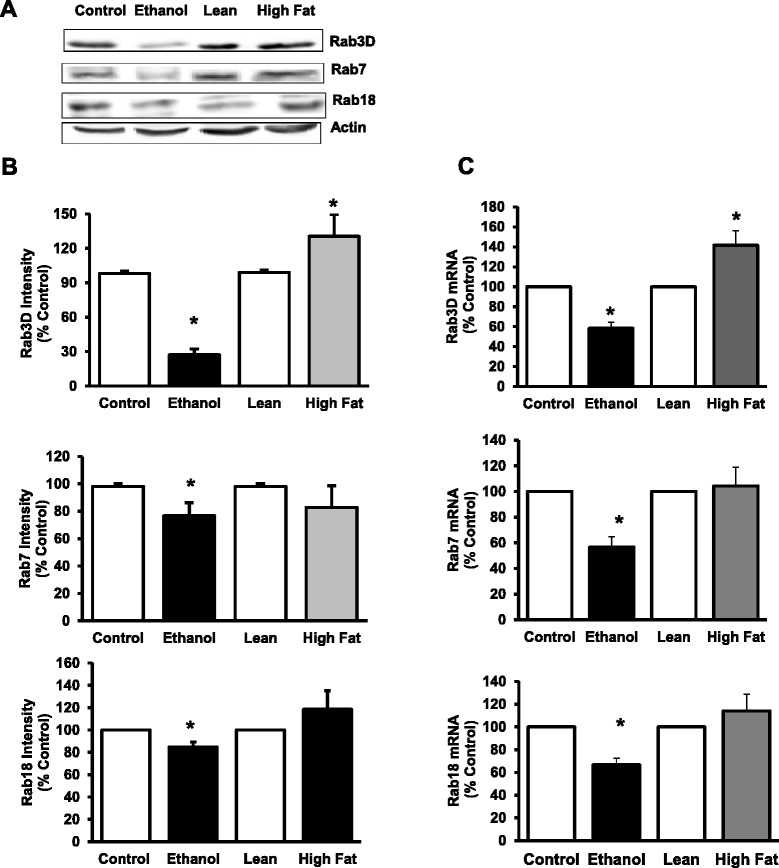
Quantification of Rab 3D, Rab 7 and Rab18 protein and mRNA content in livers of AFLD and NAFLD rats. **a** Representative Western Blot analysis for Rab3D, Rab7 and Rab18 proteins. **b** Quantitative data were analyzed as intensity units using the Odyssey Infrared Imager associated software. Results were normalized to β-actin and expressed as percentage of their respective controls. **c** mRNA expression of Rab GTPases 3D, 7 and Rab18. The mRNA levels of Rabs were measured by real-time PCR. Values represent the fold change compared to respective controls. Significant decreases in protein content and expression were noted in Rabs 3D, 7 and Rab18 for the AFLD, but not the NAFLD rats. Values are means ± SEM, *n* =8, *p ≤ 0.05

In previous studies, we have identified altered protein content for several Rab GTPases in the alcoholic fatty liver [9]. In particular, we identified decreased content for Rab3D (involved in exocytosis), Rab7 (involved in endocytosis/autophagy) and Rab18 (involved in Golgi-endoplasmic reticulum transfer). In the current study, we measured content of these proteins in livers of AFLD and NAFLD animals (Fig. 6a). Similar to what we have previously identified, ethanol treatment significantly decreased the content of Rab3D (75 %), Rab7 (25 %) and Rab18 (16 %) in liver (Fig. 6b). mRNA for Rab3D, Rab7 and Rab18 were also significantly impaired in the alcohol-fed, but not the high fat, non-alcoholic animals (Fig. 6c). In contrast, HFD animals in the NAFLD model did not show any significant change in Rab7 and Rab18, and increased Rab3D content. The mRNA profile for the NAFLD animals reflected the protein expression (Fig. 6c), and was unchanged.

### Oxidant stress and anti-oxidant defenses

**Fig. 7:**
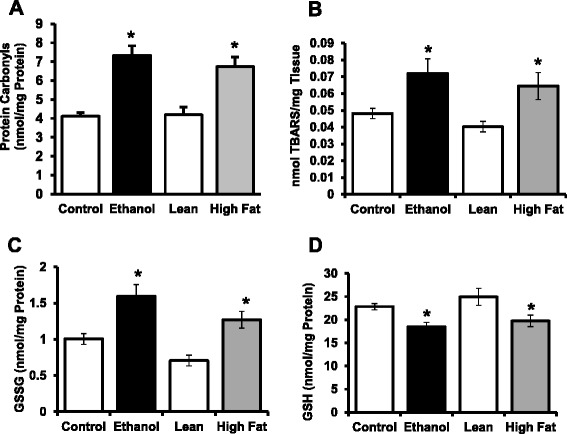
Quantification of selected parameters of oxidative stress and anti-oxidant defenses in livers of AFLD and NAFLD rats. **a** Protein carbonyls; (**b**) TBARS; (**c**) Oxidized GSH (GSSG) (**d**) Reduced GSH levels were determined as indicated in the *Methods* section. Values are means ± SEM, *n* =8, * *p* ≤ 0.05 (compared to their respective controls)

Oxidative stress and lipotoxicity are known to occur in NAFLD [29] and AFLD [30]. In regards to the oxidative stress in both comparative model systems, we observed a significant increase in protein carbonyls content, TBARS (a by-product of lipid peroxidation) and oxidized glutathione (GSSG) in livers of both ethanol-fed and high-fed animals compared to their respective control-fed animals (Fig. 7a–d). In contrast, ~25 % lower content of reduced glutathione (GSH) in livers of both EtOH-fed and HFD rats compared to their controls was observed.

## Discussion

One common classical effect of both chronic alcohol intake and high-fat diet consumption is the presence of a fatty liver, with similar histology (essentially indistinguishable) between the two types. With the increased incidence of NAFLD in the general population, it will be important to elucidate differences in mechanisms of fatty liver pathogenesis between fatty liver induced by alcohol administration (AFLD) as compared to that observed under nutrient-induced high-fat diet conditions. In this article, we have demonstrated the characteristic differences of AFLD and NAFLD regarding selected metabolic parameters and important aspects of membrane trafficking by focusing on some well-identified defects which have been involved in AFLD, and compared these results with a model of diet-induced fatty liver.

For studies reported here, Wistar rats were administered either alcohol (as 36 % of calories as ethanol, 35 % as fat, 11 % carbohydrate) in a nutritionally adequate liquid diet or a high fat diet (60 % of calories from fat, 20 % calories from carbohydrate) for 8 weeks. Thus, the composition of diet and the calorie intake are different, but both are well accepted models to induce AFLD and NAFLD respectively. Moreover, it has been shown that feeding alcohol (35 %) containing or high fat-fed diets led to histopathological hepatic changes similar to human AFLD and NAFLD [31, 32]. The mechanisms proposed to play a role in the development of alcoholic fatty liver involve an increase in the fatty acid synthesis, decrease in fatty acid oxidation and impaired VLDL secretion [33–36]. In high fat-fed conditions, fatty liver is believed to follow from the development of insulin resistance, an imbalance between hepatic lipid intake, synthesis, degradation and secretion [37, 38]. Despite the differences in underlying mechanisms, the pathological features are the same in both conditions. Thus, we took an interesting approach to elucidate the metabolic differences and similarities between these diseases. As expected, we observed increased hepatic lipid deposition in both models and the degree and distribution of lipid droplets were similar. Further assessment of hepatic function through the measure of serum enzymes and metabolites revealed both models showed significantly higher ALT and ALP when compared to their respective controls. These increases are expected indicators of liver damage. AST levels were not measured with the VetScan chemistry rotor and are not a reliable marker in simple steatohepatitis [39]. Serum bile acids were significantly increased in the ethanol-fed AFLD, but not the high-fat NAFLD animals when compared to their respective controls. An increase in serum bile acids for the AFLD rats suggest an intrahepatic cholestasis, similar to what has been reported for serum profiles of ASH and NASH patients [40–42].

Extra hepatic hormone factors are also known to critically modulate hepatic lipid metabolism. In this study, we measured insulin, amylin and leptin, all of which are known to participate in the complex process of energy homeostasis. In ethanol-fed animals, the pancreatic hormones, insulin and amylin, were found to be significantly decreased compared to the pair-fed controls. Levels of the adipocyte hormone, leptin, were not altered in sera of EtOH-fed animals. The decreased plasma levels of pancreatic hormones (insulin and amylin) likely indicate impaired pancreatic function in the setting of AFLD. In contrast, NAFLD rats fed with the HFD showed hyperinsulinemia and increased leptin levels compared to the lean controls. Amylin levels were unchanged between experimental and control animals of the NAFLD group. Hyperinsulinemia and insulin resistance are common in both diabetes and obesity, and in animal models, Samuel et al. reported that the ability of insulin to suppress hepatic glucose production is diminished in rats feeding with HFD for 3 days [43]. Thus, impaired insulin function could lead to compensatory hyperinsulinemia, where the body attempts to balance the reduced effect of insulin by producing and releasing more insulin. Along with hyperinsulinemia, we also observed increased leptin levels in NAFLD, which is likely due to leptin resistance [44, 45].

The mechanisms for progression of alcoholic liver diseases are likely multifactorial and include multiple mechanisms, with impaired protein trafficking as one likely mechanism. Dr. Casey and her laboratory have studied the events of impaired RME and its consequences in progression of liver disease by using the hepatocyte specific ASGPR. Chronic ethanol administration has been shown to alter multiple aspects of the hepatic RME pathway, including decreases in receptor expression, protein content, ligand binding, ligand internalization and receptor recycling [10, 15, 46, 47]. These impairments were identified as early as one week of ethanol administration, and were most prominent in hepatocytes isolated from the centrilobular region of the liver [48]. Consistent with those previous studies, we report here that ethanol administration showed significant impairments of ^125^I-ASOR ligand binding and internalization in hepatocytes from EtOH-fed animals when compared to controls. In parallel to decreased binding, we also observed decreases in ASGPR expression and its content in ethanol-fed rats. In contrast, no difference in binding or internalization was found in the hepatocytes isolated from the lean and high-fat animals, indicating specific alcohol-related impairments to endocytosis by the ASGPR.

In addition to endocytosis by the ASGPR, we have recently reported that ethanol administration results in decreased protein levels of several small GTPases known to play an essential role in controlling membrane trafficking of endo- and exocytic pathways [9]. Importantly, we showed that protein content of three Rabs known to be involved in vesicle trafficking, endocytosis and Golgi-endoplasmic reticulum transport (Rab3D, Rab7 and Rab 18) were are decreased in after alcohol administration, while others (Rab 1, 2 and 5) were unaffected. In the present study we had a particular interest in the Rabs which were sensitive to alcohol administration, and here, we show that in the AFLD animals, there was a marked, 75 % decrease in the content of Rab3D GTPase in liver, along with a significant decrease in Rab3D mRNA. Similarly, protein and mRNA content of Rab7 were also significantly lower in ethanol-fed, but not HFD animals, and Rab18 content and mRNA showed similar results. None of these proteins showed significant differences between the NAFLD groups. All of these Rabs could play a direct role ASGPR trafficking, which was impaired in AFLD, but not NAFLD rats. Since increased fat in the form of lipid droplets in AFLD hepatocytes arise from ethanol and acetaldehyde metabolism, it is possible that these key metabolites play a role in altered Rab function. The appearance of fatty liver in AFLD is in contrast to NAFLD hepatocytes which accumulate lipid due to mass action of bulk lipid availability. In simple steatosis without inflammation, which is portrayed by our NAFLD animal model, the liver is still relatively healthy and just beginning to show injury as measured with the ALP and ALT levels. In this state, the liver may serve as a secondary site for lipid storage due to the metabolism of high concentrations of free fatty acids absorbed by the small intestine, packaged into chylomicrons, and delivered in the lymphatics and blood stream and, ultimately, to the liver. The trafficking machinery in hepatocytes is still functional as evidenced by the normal levels of Rabs and ASGPR activity. We hypothesize that vesicle trafficking in and out of the cell is normal in NAFLD, but liposome autophagy and trafficking is impaired due to the toxic effects of ethanol metabolism to the cellular machinery in AFLD. Follow-up experimentation examining lipid-droplet trafficking in the NAFLD and how that is compared with what is known in AFLD is currently under investigation.

These findings suggest that even though both AFLD and NAFLD show the same characteristics of fat accumulation and selected examined parameters of liver pathology (including increased oxidative stress in both AFLD and NAFLD models), the impairments in membrane trafficking in the ethanol fed animals is likely a result of alcohol administration, and not from these observed changes in high-fat or EtOH-fed rats. In EtOH-fed conditions, ethanol metabolism, in addition to promoting fatty acid synthesis, also generates highly reactive acetaldehyde and reactive oxygen species (ROS) by alcohol dehydrogenase (ADH) and CYP2E1 (cytochrome P450, family 2, subfamily E, polypeptide 1) [49]. Since we observed similar changes in the selected parameters of oxidant stress and anti-oxidant defenses irrespective of the insult to the livers that resulted in fatty liver, it is likely that the observed differences we have identified are related to metabolites of alcohol. Indeed, previous work has shown in cultured cells that when alcohol metabolism is blocked by the addition of pyrazole, impaired endocytosis by the hepatic ASGPR required ethanol oxidation [50]. Additionally, our previous work showed that in the presence of an inhibitor of aldehyde dehydrogenase (cyanamide), that the impairments to endocytosis were attenuated [51].

## Conclusion

We provide evidence that the observed endocytosis and vesicle protein content in AFLD animals are most likely effects of ethanol metabolism in the liver which is not seen in NAFLD.

## Abbreviations

AFLD: alcoholic fatty liver disease
ALP: alkaline phosphatase
ALT: alanine aminotransferase
ASGPR: asialoglycoprotein receptor
ASOR: asiaoloorosomucoid
AST: aspartate aminotransferase
EtOH: ethanol
GSH: reduced glutathione
GSSG: oxidized glutathione
HFD: high fat diet
LD: lipid droplet
MDA: malondialdehyde
NAFLD: non-alcoholic fatty liver disease
RME: receptor mediated endocytosis
TBARS: thiobarbituric acid reactive substances
